# A preparation approach of exploring cluster ion implantation: from ultra-thin carbon film to graphene

**DOI:** 10.1186/1556-276X-9-205

**Published:** 2014-05-02

**Authors:** Zesong Wang, Zaodi Zhang, Rui Zhang, Hui Li, Dejun Fu

**Affiliations:** 1 Key Laboratory of Artificial Micro- and Nano-Materials of Ministry of Education of China, School of Physics and Technology, Wuhan University, Wuhan 430072, China

**Keywords:** Carbon cluster, Low-energy implantation, Graphene, Raman spectra, 29.20.-c, 29.25.Ni, 81.05.-t

## Abstract

Based on the extensive application of 2 × 1.7MV Tandetron accelerator, a low-energy cluster chamber has been built to explore for synthesizing graphene. Raman spectrum and atomic force microscopy (AFM) show that an amorphous carbon film in nanometer was deposited on the silicon by C_4_ cluster implantation. And we replaced the substrate with Ni/SiO_2_/Si and measured the thickness of Ni film by Rutherford backscattering spectrometry (RBS). Combined with suitable anneal conditions, these samples implanted by various small carbon clusters were made to grow graphene. Results from Raman spectrum reveal that few-layer graphene were obtained and discuss whether *I*_G_/*I*_2D_ can contribute to explain the relationship between the number of graphene layers and cluster implantation dosage.

## Background

In the past of several decades, ion beam analysis (IBA) based on low-energy accelerator has developed to be a comprehensive particle analytical discipline system [1-4]. A further exploitation of what can be paid more attention has springed up on the functional materials [5], *in situ* observation for defects on semiconductor industry and the simulation of multi-ion irradiation environment. For instance, the energetic ion-solid interaction was taken as a classic model to characterize some structure information of superconductor at room temperature or high K by projecting MeV ions to impact on superconductive targets [6]. In order to understand the influence induced by implanting multi-energy ions to the substrate, in particular several defects that lead to some phase transitions in matter, *in situ* characterization of these transients which can exhibit a clear physical image on changeable process of the structure was performed by the accelerator-transmission electron microscopy (TEM) interface system [7,8]. For practical application of multi-particle irradiation, the purpose of fabricating the multi-ion irradiation stage associated with simulation of the realistic environment where some special materials or functional devices are used is scientific and effective [9,10]. In a way, not only can ion beam analysis take full advantage of probing the stoichiometry but can also trace reasonable explanation on structure details of the matter [11].

In Wuhan University, the double 1.7 MV Tandetron accelerator was inherited from Physical Institution of Chinese Academy of Sciences in 2004. After several important maintenances and upgrades of facility, some primary ion beam analysis with terminal voltage at 1.2 MV can be performed in a good state, such as Rutherford backscattering spectrometry (RBS), elastic recoil detection analysis (ERDA), and nuclear reaction analysis (NRA). Besides, we have developed some extensive applications, including accelerator-TEM interface system [7] and double-ion beam radiation chamber and another new design of low-energy cluster chamber for ion implantation.

As another kind of ultra-thin carbon film, graphene is a promising material which is probable to replace silicon integration technique due to its advanced and novel physical properties [12,13]. But a key issue for preparing larger scale and continuous thin film is always not comprehensively figured out. Garaj et al. and Baraton et al. have reported graphene synthesis by ion implantation at 30 keV [14] and 80 keV [15], respectively. But cluster ions have not been involved, especially in the case of lower energy implantation. Therefore, it is a reasonable attempt that can be attributed to much shallower penetration depth from low-energy cluster ions to dedicate to carbon atoms precipitation form the transition metal under subsequent thermal treatments. In this work, above low-energy cluster chamber is addressed to synthesis nanostructure carbon materials including ultra-thin film and graphene, expanding fundamental ion beam applications in this machine.

## Methods

### Low-energy cluster chamber

A source of negative ion by cesium sputtering (SNICS) can produce various negative ions from solid targets, such as B^−^, C^−^, Si^−^, P^−^, Fe^−^, Cu^−^, and Au^−^[16,17], which can be implanted into the substrates after being accelerated up to the maximum 30 keV depending on the accelerator field. Selecting cluster ions with small size as projectiles to perform the process of low-energy ion implantation can form shallow layer architectures in the matrix, which is beneficial to fabricate ultra-shallow junction devices.

**Figure 1:**
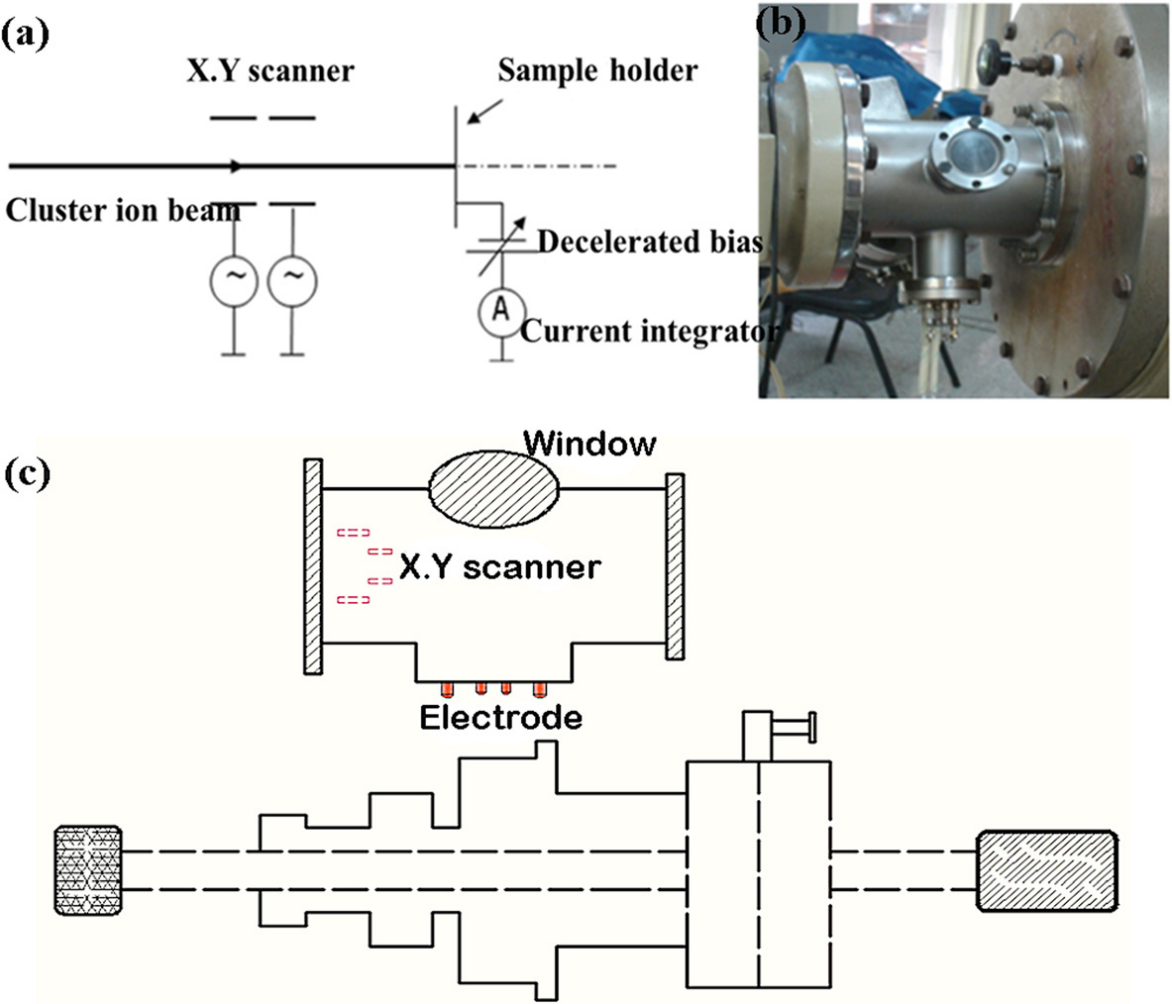
**Schematic diagram of low-energy cluster deposition. (a)** The schematic diagram of cluster ion deposition. **(b)** The graph of deposition in chamber. **(c)** Top view of chamber and the rotated sample holder.

Figure 1a,b illustrates the schematic diagram of low-energy cluster deposition. In our previous study [18], some carbon cluster ions (Cn^−^) from SNICS at an energy of 20 keV are chosen for desirable targets by mass analyzer, then are decelerated to a few hundred electron volt or below 3 keV by the deceleration field after voltage scanner mounted on two aligned directions of *X* and *Y*-axis, finally to soft-land to the substrate. The current integrator is used for monitoring implantation dose simultaneously. To eliminate some impacts on the current integrator from high voltage at decelerated filed, an isolation transformer was introduced to guarantee safety. In addition, a rotated target holder (Figure 1c) was designed to change projectile ranges of cluster ions by regulating the angle between incident ion and the substrate. The overall layout, similar to ion beam-assisted deposition, was executed to deposit carbon cluster ions onto the surface of silicon for graphene synthesis. Unfortunately, it is not successful to obtain graphene for this method. However, some ultra-thin carbon films on the silicon were prepared with the scale of several nanometers.

## Results and discussion

### Ultra-thin carbon film deposition

**Figure 2:**
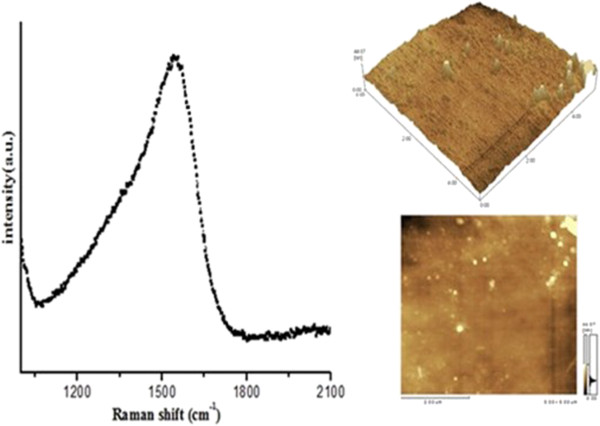
**Raman spectra and AFM image of the sample by C**_**4 **_**cluster ion implantation.**

Figure 2 shows Raman spectrum and atomic force microscopy (AFM) images of the sample synthesized by C_4_ ions implantation. The projectile range of C_4_ in the silicon is approximately 5 nm at 14 keV, which was calculated by SRIM 2008 edition [19]. About 12 kV terminal voltages were reversed to slow down the kinetic energy of cluster ions so as to soft-land onto the surface of the sample. Expectedly, such low-energy interactions of cluster target can lead to little cascade collisions. Raman spectra indicate that there is an amorphous carbon film on the sample due to sp^2^ hybridized carbon atoms forming *π*-bond to enhance Raman scattering cross section, which is performing drastic peak intensities at about 1,560 cm^−1^. In conjunction with the surface morphology of AFM image, the amorphous layer exhibits continuous distributions on the whole substrate except some possible island-like contaminations in the form of white spots. Certainly, these columnar protuberances may be some larger grain accumulations induced by higher energetic ions landing on the edge than that in the center of the sample, depending on the strength distribution of decelerated field. The value of root mean square roughness (RMS) is about 5.10 nm for thin film, which indicates a great promise of preparing ultra-thin film under much lower energy ion implantation.

### Few-layer graphene synthesis

It is an essential purpose that we designed this low-energy cluster chamber for graphene preparation. In the process of exploring some effective methods, after depositing carbon films with the scale of several nanometers on the silicon, we selected suitable substrates to succeed in achieving few-layer graphene. Uninstalling the decelerated field, we selected small carbon cluster ions to inject to the substrate below 30 keV. The substrate Ni/SiO_2_/Si with about 300 nm Ni film deposited Ni atoms onto silica by e-beam evaporating.

**Figure 3:**
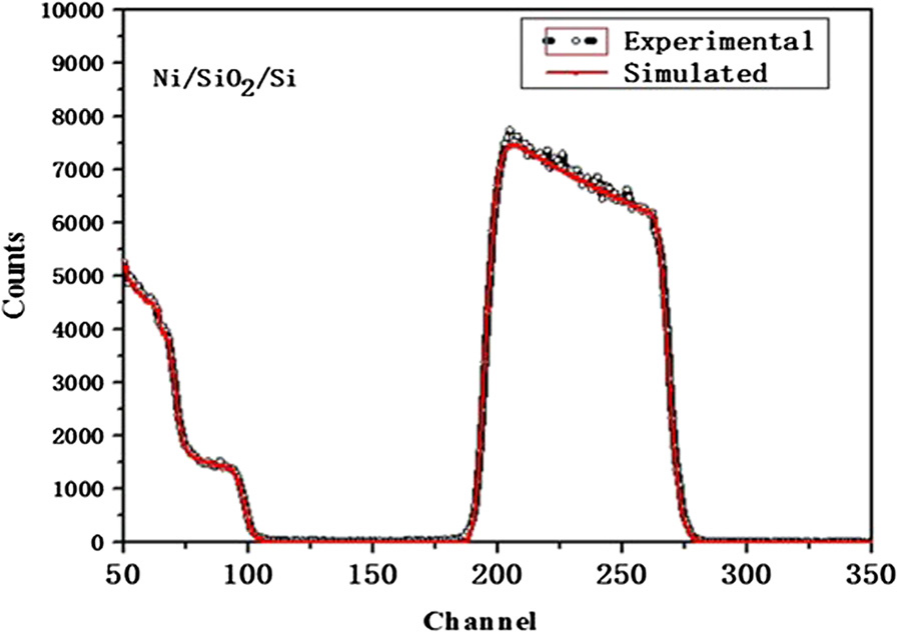
**RBS spectra of Ni/SiO2/Si with incident 2.86 MeV Li**^**2+**^**.**

The thickness of Ni film has influence on carbon segregation from inside up to the surface, so it is significant to evaluate the thickness of the substrate, and RBS spectra of the sample was carried out, as shown in Figure 3. Incident 2.86 MeV Li^2+^ which was produced by the double 1.7 MV tandem accelerator was collimated to the target with ion current of 5 nA and the round beam spot of 1.5 mm. The backscattered ions were detected by passivated implanted planar silicon (PIPS) detector with the resolution of 14 keV for *α* particle at 165°. The abscissa of spectra stands for channel numbers of multi-channel analyzer (MCA), which is proportional to the energy of scattered ions. A broad peak indicates that the surface edge of Ni is about channel 269 and the back edge is about channel 195. The channel difference of both edges is corresponding to the energy loss of projectile Li ions in Ni in correlation with the thickness of thin film. A straightforward route is simulating the trajectories of incident ions in matter. The red curve of this graph is simulation result from SIMNRA6.05 code, which is in coincidence with experimental data absolutely. The simulated results reveal that the areal density of Ni film is 2.1 × 10^18^ atoms/cm^2^, and a corresponding thickness is 227.3 nm in the case of the volume density at 8.9 g/cm^3^, which is thinner than the estimated value.

With regard to depositing Ni film onto silica but not silicon substrate, it was reported that the silicon oxide at a thickness of 300 nm can enhance scattered signals of Raman resonance spectrum drastically because photon can evoke continuous interferences at the interface between Ni and silica [20]. All the matrixes were implanted with the same dosage at 8 × 10^15^ cm^−2^ by ion implantation consisting of different cluster sizes at 20 keV. After implantation, these samples were annealed from room temperature to 900°C and dwell time was 60 min, then cooled down to room temperature naturally at 2.0 torr.

**Figure 4:**
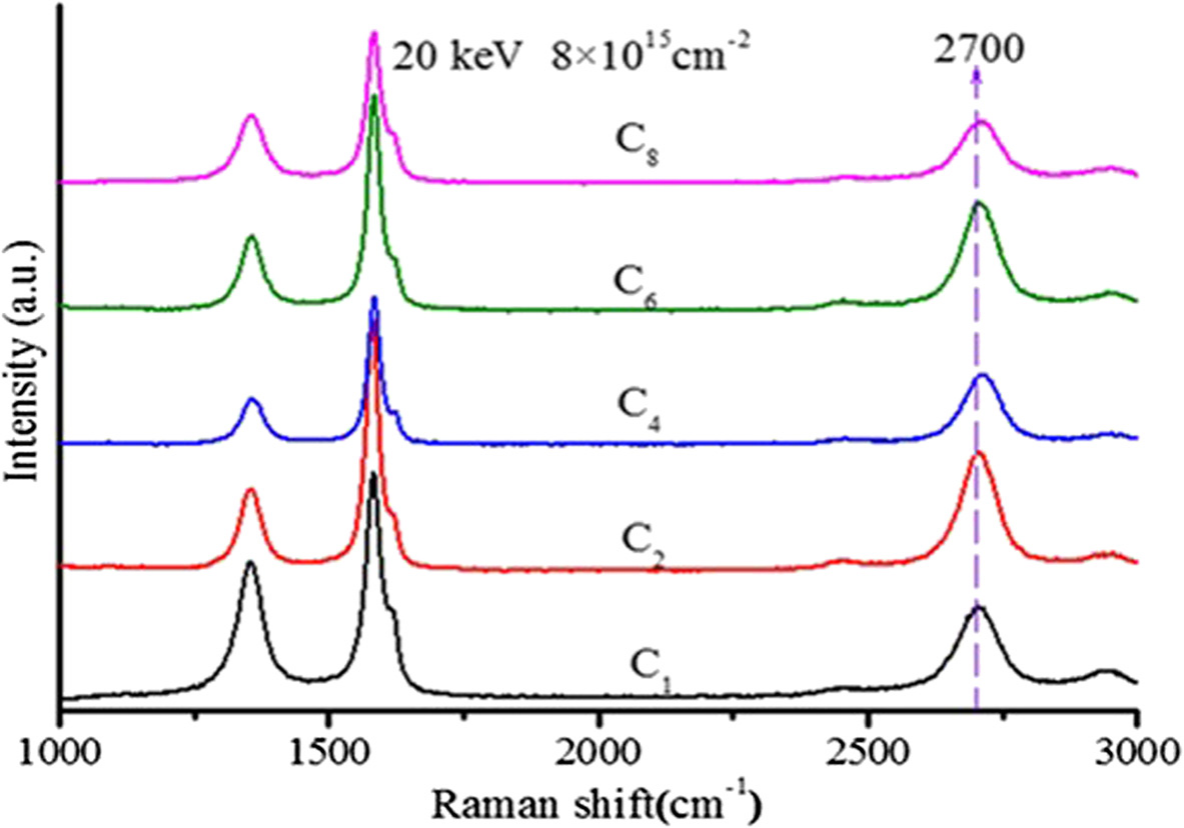
**Raman spectra of the samples implanted by the different kinds of carbon clusters C**_***n***_**(*n*** **= 1, 2, 4, 6, 8).**

**Figure 5:**
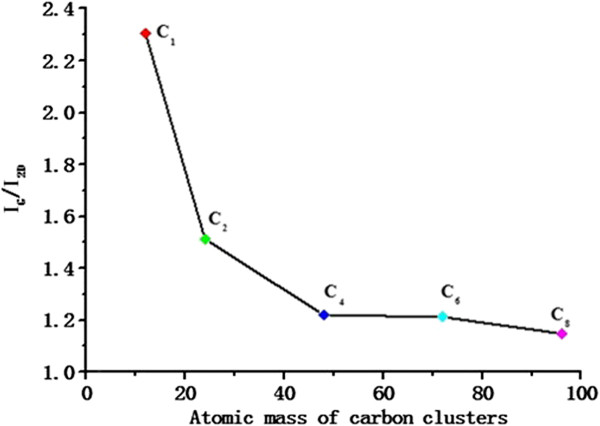
**The intensity ratio *I***_**G**_**/*I***_**2D**_**as functions of the mass small carbon cluster.**

Raman spectroscopy is always employed as one of the powerful non-destructive methods to identify graphene and determine the layer of graphene [15,21]. In this study, Raman scattering was excited by an Ar laser at 514 nm and the power at the sample is below 1 mW for avoiding radiation damage. Figure 4 shows Raman spectra of the samples. For 514-nm wavelength laser, D peak position at 1,350 cm^−1^ is relative to the disorder and defects in the structures performing sp^3^ hybridization of carbon atoms, while sp^2^ hybridization induced by the in-plan optical phonon E_2g_ near the first Brillouin Zone center is characterized as G peak at 1,580 cm^−1^[22]. The 2D peak position at 2,700 cm^−1^ of graphene is single and symmetrical to characterize monolayer. These samples were implanted with the same dosage of 8 × 10^15^ carbon atoms/cm^2^ at 20 keV by the different small carbon cluster sizes (C_1_, C_2_, C_4_, C_6_, C_8_). Almost the three characteristic peak positions appear, and every peak position for different cluster sizes has also negligible shifts, as shown in Figure 4. In most literatures, 2D peak position at 2,700 cm^−1^ and *I*_G_/*I*_2D_ (the intensity ratio of G peak and 2D peak), which is the smaller and thinner film that can be obtained, were also evaluated to differentiate graphite and confirm the layers of graphene sheets [20]. The range of 2D peak position is 2,704 to 2,709 cm^−1^ in the spectra, corresponding to three and more layers. A visualized trend is observed that *I*_G_/*I*_2D_ decreases as carbon cluster size increases, described in Figure 5. There is a drastic decline for small clusters C_1_ to C_4_, meanwhile larger clusters C_4_, C_6_, C_8_ are presenting a relatively gradual shrink. In the case of such low-energy ion implantation, light cluster can penetrate into deeper sites than heavy cluster in the substrate, which is dependent on the energy distribution of cascade collision between cluster and matter.

In previous studies, we have achieved some carbon cluster mass spectra at the different extractor voltages of sputtering ion source in this machine and paid much attention to investigate Raman characterizations of graphene by means of the carbon cluster ion implantation [18,23]. Because the cluster ion current can be influenced from cluster size and extractor bias strongly, selecting small carbon cluster ions to carry out implantation is out of more time consumptions. However, more defects can be produced by cluster C_1_ implantation instead of saving time. For example, implantation time is about 8.5 h for cluster C_8_ at 20 keV in this work, but the *I*_G_/*I*_2D_ ratio is the smallest which indicates that the graphene quality is better than that in the other smaller cluster sizes.

Simply, *E*_0_ is cluster energy, and every atom of Cn cluster can be allocated as homogeneous energy of *E*_0_/*n*. Therefore, in comparison with C_1_, C_*n*_ (*n* > 1) has more sophisticated interactions with the substrate, involving in non-linear damage effect and atomic self-sputtering effect [24,25]. During such low-energy shallow ion implantation, carbon atom contents in Ni film may reach up to saturation at certain implantation dosage, which is significant for cluster aggregation to interact with the substrate. Graphene nucleation on the transition metal has been investigated to a theoretical growth issue that strongly depends on segregation and precipitation on the grain boundaries of the substrate after thermal treatment [26], no matter how to prepare graphene, by chemical vapor deposition (CVD) or ion implantation [14,15,20,21]. Baraton et al. have proposed that the anneal temperature from 900°C to 725°C, half of the carbon atoms were removed to grain boundaries of Ni surface to form graphene; that is to say, 4 × 10^15^ cm^−2^ and 8 × 10^15^ cm^−2^ of carbon concentration on the surface are in agreement with monolayer and bilayer graphene [15], respectively. However, it is not successful to control the number of graphene layers accurately by regulating the contents of implantation carbon atoms. We always seek to graphene synthesis with fewer defects by low-energy cluster ion technique; larger cluster size C_*n*_ (*n* > 10) under suitable energy is more likely to develop this process. But we have to take the atomic self-sputtering effect and more sophisticated cluster-matter interaction into consideration. More investigations are probable to promote the nucleation mechanism of graphene including ion-matter interaction, crystal quality of the substrate, anneal temperature, and other details about growth conditions.

## Conclusions

We have developed a low-energy cluster chamber on the base of extensive application for the double 1.7 MV Tandetron accelerator, which was used to explore for graphene synthesis. In our previous work, a kind of amorphous ultra-thin carbon film was fabricated by projecting C_4_ cluster ions to the silicon at 14 keV, and the RMS is about 5.10 nm. Another substrates Ni/SiO_2_/Si whose thickness was measured at 227.3 nm by RBS were implanted with the same carbon atoms at 8 × 10^15^ cm^−2^ by several kinds of small clusters C_*n*_ (*n* = 1, 2, 4, 6, 8); after annealing, Raman spectra indicate that few-layer graphene was prepared successfully. And the ratio *I*_G_/*I*_2D_ shows that the number of graphene layers cannot be controlled by implantation dosage purely but are associated with carbon atoms precipitation and segregation from inside to the surface grain boundaries of the substrate during thermal treatment. From ultra-thin carbon film to graphene by means of the similar cluster ion implantation technique, it is conductive for cluster implantation of light elements to develop low-energy shallow ion implantation in semiconductor industry.

## Abbreviations

AFM: atomic force microscopy; ERDA: elastic recoil detection analysis; IBA: ion beam analysis; MCA: multi-channel analyzer; NRA: nuclear reaction analysis; RBS: Rutherford backscattering spectrometry; RMS: root mean square roughness; TEM: transmission electron microscopy.

## Competing interests

The authors declare that they have no competing interests.

## Authors’ contributions

ZW designed parts of the experiments and sample preparations and drafted the manuscript. DF is the corresponding author and provided a great help for experimental designs. Other co-authors took part in sample preparation and characterizations and discussed the results. All authors have read and approved the final manuscript.
